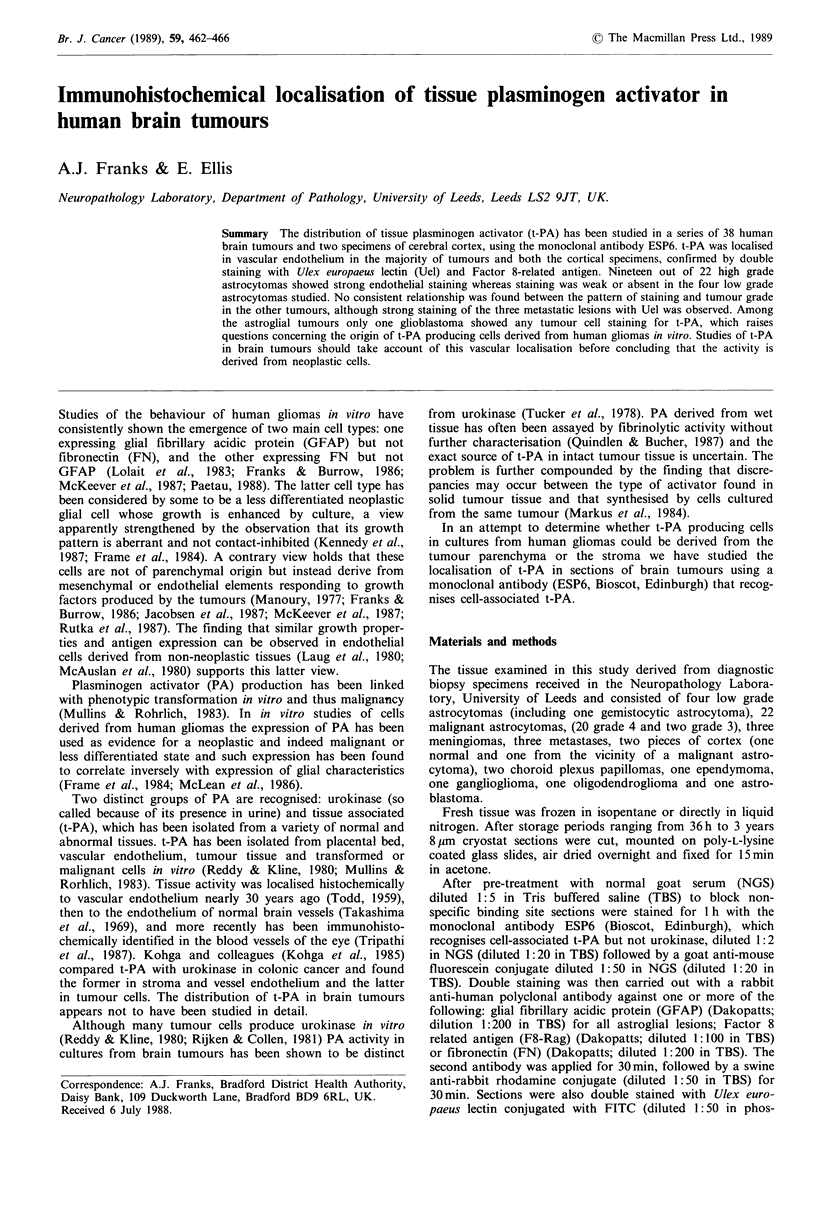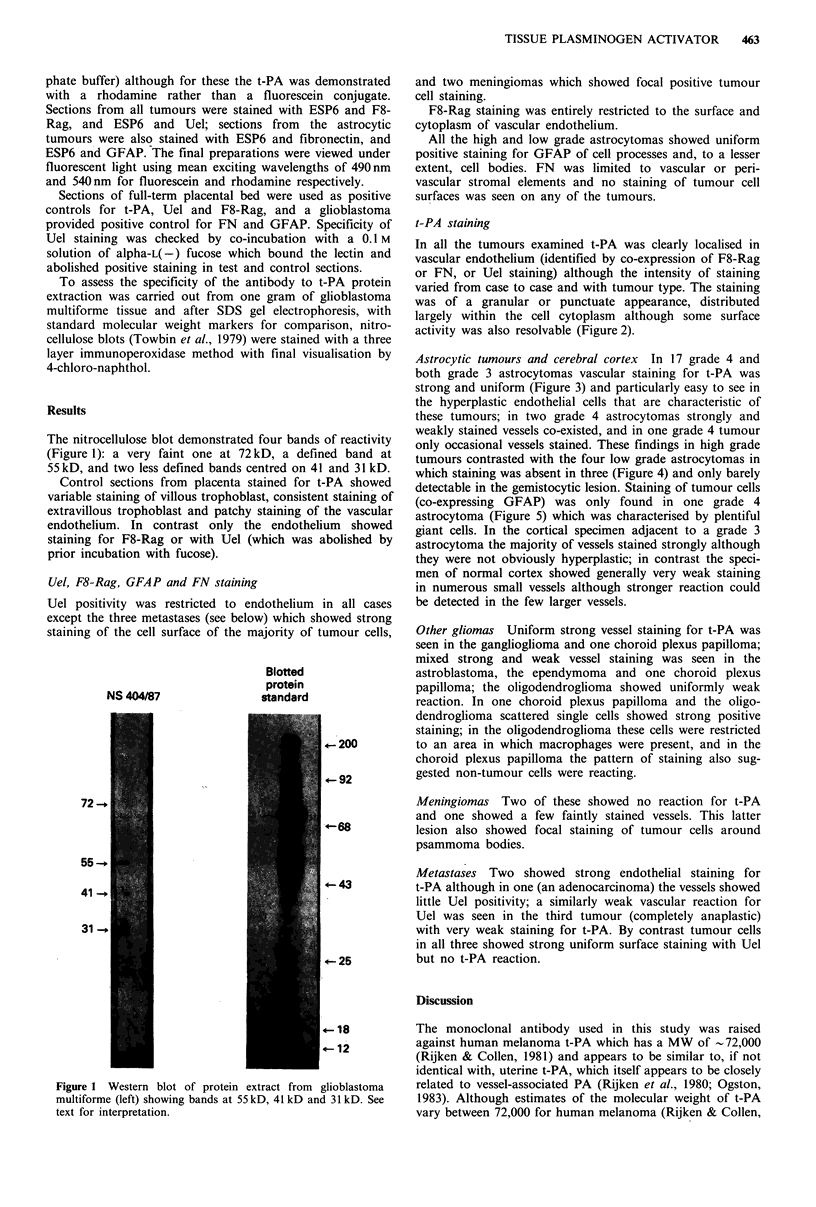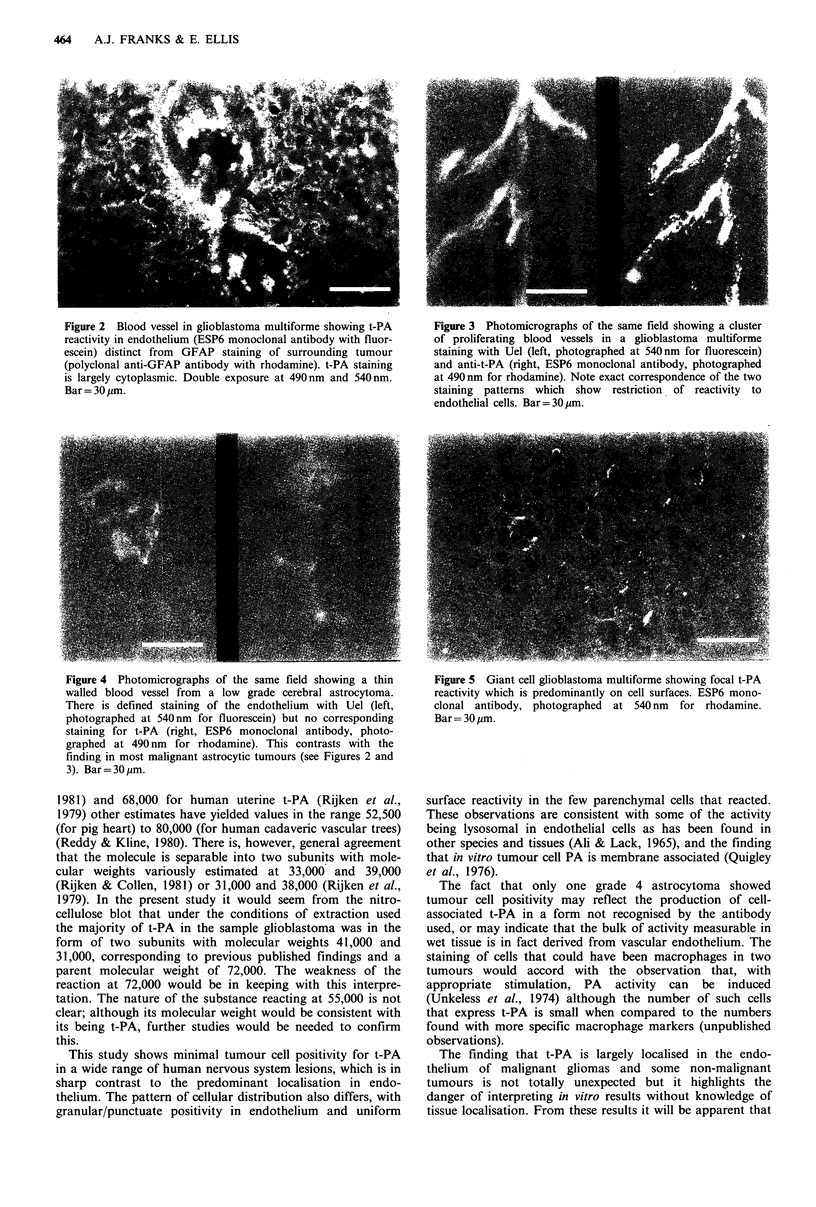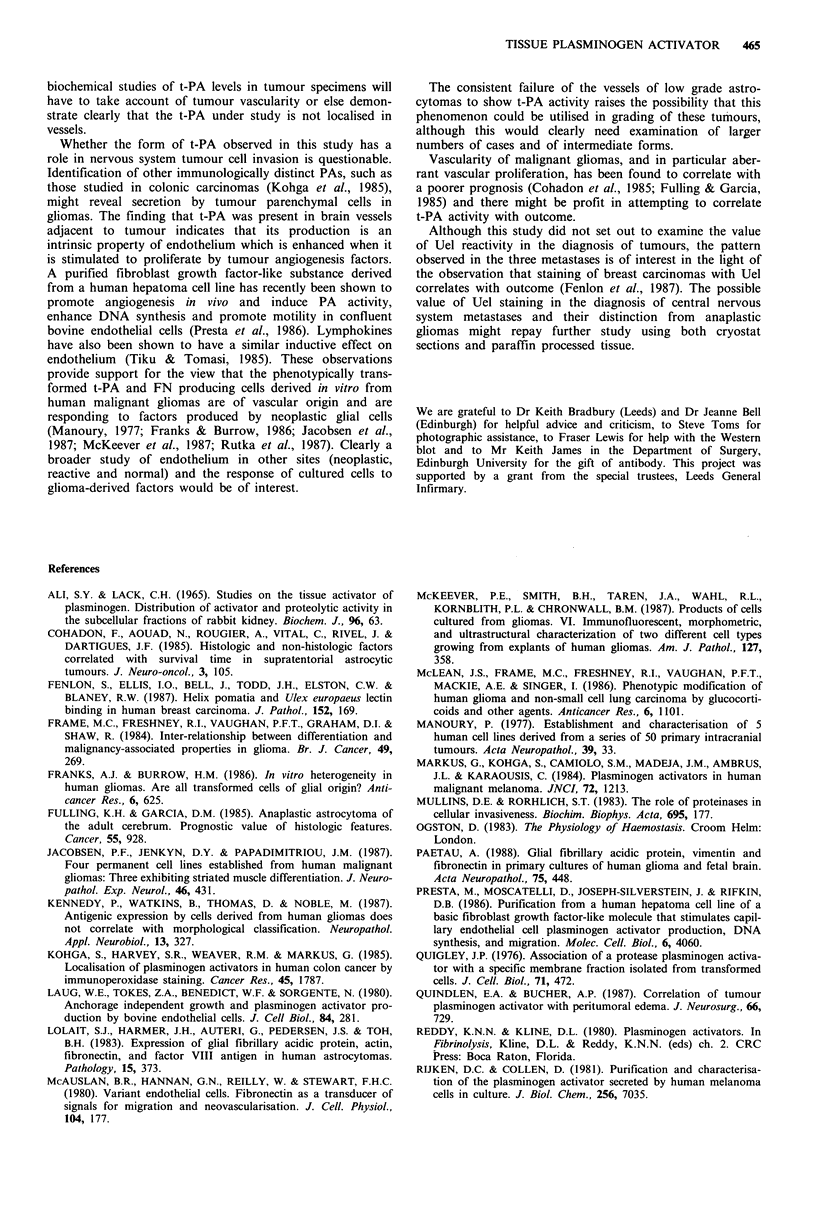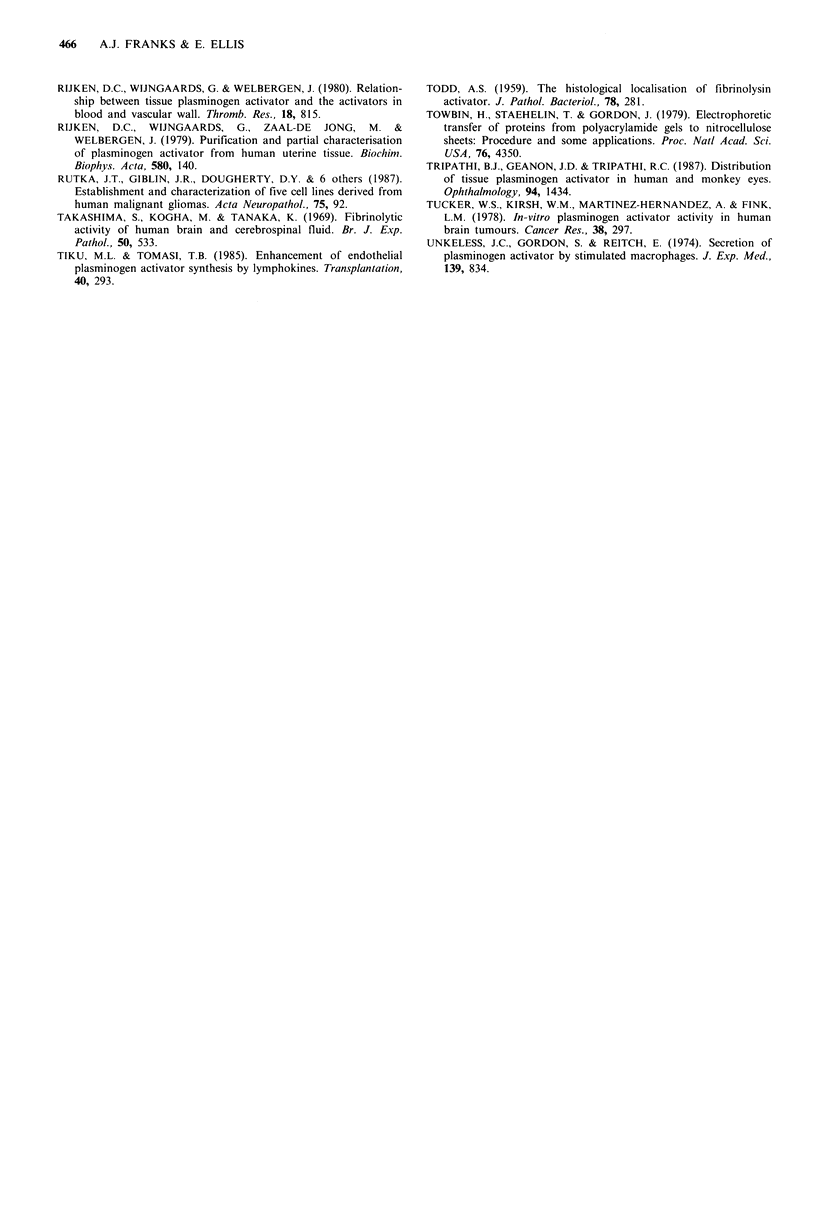# Immunohistochemical localisation of tissue plasminogen activator in human brain tumours.

**DOI:** 10.1038/bjc.1989.95

**Published:** 1989-03

**Authors:** A. J. Franks, E. Ellis

**Affiliations:** Neuropathology Laboratory, Department of Pathology, University of Leeds, UK.

## Abstract

**Images:**


*Br. J. Cancer* (1989), **59**, 462–466 © The Macmillan Press Ltd., 1989

### Immunohistochemical localisation of tissue plasminogen activator in human brain tumours

A.J. Franks & E. Ellis

*Neuropathology Laboratory, Department of Pathology, University of Leeds, Leeds LS2 9JT, UK.*

**Summary** The distribution of tissue plasminogen activator (t-PA) has been studied in a series of 38 human brain tumours and two specimens of cerebral cortex, using the monoclonal antibody ESP6. t-PA was localised in vascular endothelium in the majority of tumours and both the cortical specimens, confirmed by double staining with *Ulex europaeus* lectin (Uel) and Factor 8-related antigen. Nineteen out of 22 high grade astrocytomas showed strong endothelial staining whereas staining was weak or absent in the four low grade astrocytomas studied. No consistent relationship was found between the pattern of staining and tumour grade in the other tumours, although strong staining of the three metastatic lesions with Uel was observed. Among the astroglial tumours only one glioblastoma showed any tumour cell staining for t-PA, which raises questions concerning the origin of t-PA producing cells derived from human gliomas *in vitro*. Studies of t-PA in brain tumours should take account of this vascular localisation before concluding that the activity is derived from neoplastic cells.

Studies of the behaviour of human gliomas *in vitro* have consistently shown the emergence of two main cell types: one expressing glial fibrillary acidic protein (GFAP) but not fibronectin (FN), and the other expressing FN but not GFAP (Lolait *et al.*, 1983; Franks & Burrow, 1986; McKeever *et al.*, 1987; Paetau, 1988). The latter cell type has been considered by some to be a less differentiated neoplastic glial cell whose growth is enhanced by culture, a view apparently strengthened by the observation that its growth pattern is aberrant and not contact-inhibited (Kennedy *et al.*, 1987; Frame *et al.*, 1984). A contrary view holds that these cells are not of parenchymal origin but instead derive from mesenchymal or endothelial elements responding to growth factors produced by the tumours (Manoury, 1977; Franks & Burrow, 1986; Jacobsen *et al.*, 1987; McKeever *et al.*, 1987; Rutka *et al.*, 1987). The finding that similar growth properties and antigen expression can be observed in endothelial cells derived from non-neoplastic tissues (Laug *et al.*, 1980; McAuslan *et al.*, 1980) supports this latter view.

Plasminogen activator (PA) production has been linked with phenotypic transformation *in vitro* and thus malignancy (Mullins & Rohrlich, 1983). In *in vitro* studies of cells derived from human gliomas the expression of PA has been used as evidence for a neoplastic and indeed malignant or less differentiated state and such expression has been found to correlate inversely with expression of glial characteristics (Frame *et al.*, 1984; McLean *et al.*, 1986).

Two distinct groups of PA are recognised: urokinase (so called because of its presence in urine) and tissue associated (t-PA), which has been isolated from a variety of normal and abnormal tissues. t-PA has been isolated from placental bed, vascular endothelium, tumour tissue and transformed or malignant cells *in vitro* (Reddy & Kline, 1980; Mullins & Rorhlich, 1983). Tissue activity was localised histochemically to vascular endothelium nearly 30 years ago (Todd, 1959), then to the endothelium of normal brain vessels (Takashima *et al.*, 1969), and more recently has been immunohistochemically identified in the blood vessels of the eye (Tripathi *et al.*, 1987). Kohga and colleagues (Kohga *et al.*, 1985) compared t-PA with urokinase in colonic cancer and found the former in stroma and vessel endothelium and the latter in tumour cells. The distribution of t-PA in brain tumours appears not to have been studied in detail.

Although many tumour cells produce urokinase *in vitro* (Reddy & Kline, 1980; Rijken & Collen, 1981) PA activity in cultures from brain tumours has been shown to be distinct from urokinase (Tucker *et al.*, 1978). PA derived from wet tissue has often been assayed by fibrinolytic activity without further characterisation (Quindlen & Bucher, 1987) and the exact source of t-PA in intact tumour tissue is uncertain. The problem is further compounded by the finding that discrepancies may occur between the type of activator found in solid tumour tissue and that synthesised by cells cultured from the same tumour (Markus *et al.*, 1984).

In an attempt to determine whether t-PA producing cells in cultures from human gliomas could be derived from the tumour parenchyma or the stroma we have studied the localisation of t-PA in sections of brain tumours using a monoclonal antibody (ESP6, Bioscot, Edinburgh) that recognises cell-associated t-PA.

### Materials and methods

The tissue examined in this study derived from diagnostic biopsy specimens received in the Neuropathology Laboratory, University of Leeds and consisted of four low grade astrocytomas (including one gemistocytic astrocytoma), 22 malignant astrocytomas, (20 grade 4 and two grade 3), three meningiomas, three metastases, two pieces of cortex (one normal and one from the vicinity of a malignant astrocytoma), two choroid plexus papillomas, one ependymoma, one ganglioglioma, one oligodendroglioma and one astroblastoma.

Fresh tissue was frozen in isopentane or directly in liquid nitrogen. After storage periods ranging from 36 h to 3 years 8 $\mu$m cryostat sections were cut, mounted on poly-L-lysine coated glass slides, air dried overnight and fixed for 15 min in acetone.

After pre-treatment with normal goat serum (NGS) diluted 1:5 in Tris buffered saline (TBS) to block non-specific binding site sections were stained for 1 h with the monoclonal antibody ESP6 (Bioscot, Edinburgh), which recognises cell-associated t-PA but not urokinase, diluted 1:2 in NGS (diluted 1:20 in TBS) followed by a goat anti-mouse fluorescein conjugate diluted 1:50 in NGS (diluted 1:20 in TBS). Double staining was then carried out with a rabbit anti-human polyclonal antibody against one or more of the following: glial fibrillary acidic protein (GFAP) (Dakopatts; dilution 1:200 in TBS) for all astroglial lesions; Factor 8 related antigen (F8-Rag) (Dakopatts; diluted 1:100 in TBS) or fibronectin (FN) (Dakopatts; diluted 1:200 in TBS). The second antibody was applied for 30 min, followed by a swine anti-rabbit rhodamine conjugate (diluted 1:50 in TBS) for 30 min. Sections were also double stained with *Ulex europaeus* lectin conjugated with FITC (diluted 1:50 in phos-

Correspondence: A.J. Franks, Bradford District Health Authority, Daisy Bank, 109 Duckworth Lane, Bradford BD9 6RL, UK.
Received 6 July 1988.

phate buffer) although for these the t-PA was demonstrated with a rhodamine rather than a fluorescein conjugate. Sections from all tumours were stained with ESP6 and F8-Rag, and ESP6 and Uel; sections from the astrocytic tumours were also stained with ESP6 and fibronectin, and ESP6 and GFAP. The final preparations were viewed under fluorescent light using mean exciting wavelengths of 490 nm and 540 nm for fluorescein and rhodamine respectively.

Sections of full-term placental bed were used as positive controls for t-PA, Uel and F8-Rag, and a glioblastoma provided positive control for FN and GFAP. Specificity of Uel staining was checked by co-incubation with a 0.1 M solution of alpha-L(−) fucose which bound the lectin and abolished positive staining in test and control sections.

To assess the specificity of the antibody to t-PA protein extraction was carried out from one gram of glioblastoma multiforme tissue and after SDS gel electrophoresis, with standard molecular weight markers for comparison, nitrocellulose blots (Towbin *et al.*, 1979) were stained with a three layer immunoperoxidase method with final visualisation by 4-chloro-naphthol.

### Results

The nitrocellulose blot demonstrated four bands of reactivity (Figure 1): a very faint one at 72 kD, a defined band at 55 kD, and two less defined bands centred on 41 and 31 kD.

Control sections from placenta stained for t-PA showed variable staining of villous trophoblast, consistent staining of extravillous trophoblast and patchy staining of the vascular endothelium. In contrast only the endothelium showed staining for F8-Rag or with Uel (which was abolished by prior incubation with fucose).

### *Uel, F8-Rag, GFAP and FN staining*

Uel positivity was restricted to endothelium in all cases except the three metastases (see below) which showed strong staining of the cell surface of the majority of tumour cells,

**Figure 1** Western blot of protein extract from glioblastoma multiforme (left) showing bands at 55 kD, 41 kD and 31 kD. See text for interpretation.

and two meningiomas which showed focal positive tumour cell staining.

F8-Rag staining was entirely restricted to the surface and cytoplasm of vascular endothelium.

All the high and low grade astrocytomas showed uniform positive staining for GFAP of cell processes and, to a lesser extent, cell bodies. FN was limited to vascular or perivascular stromal elements and no staining of tumour cell surfaces was seen on any of the tumours.

### *t-PA staining*

In all the tumours examined t-PA was clearly localised in vascular endothelium (identified by co-expression of F8-Rag or FN, or Uel staining) although the intensity of staining varied from case to case and with tumour type. The staining was of a granular or punctuate appearance, distributed largely within the cell cytoplasm although some surface activity was also resolvable (Figure 2).

*Astrocytic tumours and cerebral cortex* In 17 grade 4 and both grade 3 astrocytomas vascular staining for t-PA was strong and uniform (Figure 3) and particularly easy to see in the hyperplastic endothelial cells that are characteristic of these tumours; in two grade 4 astrocytomas strongly and weakly stained vessels co-existed, and in one grade 4 tumour only occasional vessels stained. These findings in high grade tumours contrasted with the four low grade astrocytomas in which staining was absent in three (Figure 4) and only barely detectable in the gemistocytic lesion. Staining of tumour cells (co-expressing GFAP) was only found in one grade 4 astrocytoma (Figure 5) which was characterised by plentiful giant cells. In the cortical specimen adjacent to a grade 3 astrocytoma the majority of vessels stained strongly although they were not obviously hyperplastic; in contrast the specimen of normal cortex showed generally very weak staining in numerous small vessels although stronger reaction could be detected in the few larger vessels.

*Other gliomas* Uniform strong vessel staining for t-PA was seen in the ganglioglioma and one choroid plexus papilloma; mixed strong and weak vessel staining was seen in the astroblastoma, the ependymoma and one choroid plexus papilloma; the oligodendroglioma showed uniformly weak reaction. In one choroid plexus papilloma and the oligodendroglioma scattered single cells showed strong positive staining; in the oligodendroglioma these cells were restricted to an area in which macrophages were present, and in the choroid plexus papilloma the pattern of staining also suggested non-tumour cells were reacting.

*Meningiomas* Two of these showed no reaction for t-PA and one showed a few faintly stained vessels. This latter lesion also showed focal staining of tumour cells around psammoma bodies.

*Metastases* Two showed strong endothelial staining for t-PA although in one (an adenocarcinoma) the vessels showed little Uel positivity; a similarly weak vascular reaction for Uel was seen in the third tumour (completely anaplastic) with very weak staining for t-PA. By contrast tumour cells in all three showed strong uniform surface staining with Uel but no t-PA reaction.

### Discussion

The monoclonal antibody used in this study was raised against human melanoma t-PA which has a MW of ~72,000 (Rijken & Collen, 1981) and appears to be similar to, if not identical with, uterine t-PA, which itself appears to be closely related to vessel-associated PA (Rijken *et al.*, 1980; Ogston, 1983). Although estimates of the molecular weight of t-PA vary between 72,000 for human melanoma (Rijken & Collen,

**Figure 2** Blood vessel in glioblastoma multiforme showing t-PA reactivity in endothelium (ESP6 monoclonal antibody with fluorescein) distinct from GFAP staining of surrounding tumour (polyclonal anti-GFAP antibody with rhodamine). t-PA staining is largely cytoplasmic. Double exposure at 490 nm and 540 nm. Bar = 30 μm.

**Figure 3** Photomicrographs of the same field showing a cluster of proliferating blood vessels in a glioblastoma multiforme staining with Uel (left, photographed at 540 nm for fluorescein) and anti-t-PA (right, ESP6 monoclonal antibody, photographed at 490 nm for rhodamine). Note exact correspondence of the two staining patterns which show restriction of reactivity to endothelial cells. Bar = 30 μm.

**Figure 4** Photomicrographs of the same field showing a thin walled blood vessel from a low grade cerebral astrocytoma. There is defined staining of the endothelium with Uel (left, photographed at 540 nm for fluorescein) but no corresponding staining for t-PA (right, ESP6 monoclonal antibody, photographed at 490 nm for rhodamine). This contrasts with the finding in most malignant astrocytic tumours (see Figures 2 and 3). Bar = 30 μm.

**Figure 5** Giant cell glioblastoma multiforme showing focal t-PA reactivity which is predominantly on cell surfaces. ESP6 monoclonal antibody, photographed at 540 nm for rhodamine. Bar = 30 μm.

1981) and 68,000 for human uterine t-PA (Rijken *et al.*, 1979) other estimates have yielded values in the range 52,500 (for pig heart) to 80,000 (for human cadaveric vascular trees) (Reddy & Kline, 1980). There is, however, general agreement that the molecule is separable into two subunits with molecular weights variously estimated at 33,000 and 39,000 (Rijken & Collen, 1981) or 31,000 and 38,000 (Rijken *et al.*, 1979). In the present study it would seem from the nitrocellulose blot that under the conditions of extraction used the majority of t-PA in the sample glioblastoma was in the form of two subunits with molecular weights 41,000 and 31,000, corresponding to previous published findings and a parent molecular weight of 72,000. The weakness of the reaction at 72,000 would be in keeping with this interpretation. The nature of the substance reacting at 55,000 is not clear; although its molecular weight would be consistent with its being t-PA, further studies would be needed to confirm this.

This study shows minimal tumour cell positivity for t-PA in a wide range of human nervous system lesions, which is in sharp contrast to the predominant localisation in endothelium. The pattern of cellular distribution also differs, with granular/punctuate positivity in endothelium and uniform surface reactivity in the few parenchymal cells that reacted. These observations are consistent with some of the activity being lysosomal in endothelial cells as has been found in other species and tissues (Ali & Lack, 1965), and the finding that *in vitro* tumour cell PA is membrane associated (Quigley *et al.*, 1976).

The fact that only one grade 4 astrocytoma showed tumour cell positivity may reflect the production of cell-associated t-PA in a form not recognised by the antibody used, or may indicate that the bulk of activity measurable in wet tissue is in fact derived from vascular endothelium. The staining of cells that could have been macrophages in two tumours would accord with the observation that, with appropriate stimulation, PA activity can be induced (Unkeless *et al.*, 1974) although the number of such cells that express t-PA is small when compared to the numbers found with more specific macrophage markers (unpublished observations).

The finding that t-PA is largely localised in the endothelium of malignant gliomas and some non-malignant tumours is not totally unexpected but it highlights the danger of interpreting *in vitro* results without knowledge of tissue localisation. From these results it will be apparent that biochemical studies of t-PA levels in tumour specimens will have to take account of tumour vascularity or else demonstrate clearly that the t-PA under study is not localised in vessels.

Whether the form of t-PA observed in this study has a role in nervous system tumour cell invasion is questionable. Identification of other immunologically distinct PAs, such as those studied in colonic carcinomas (Kohga *et al*., 1985), might reveal secretion by tumour parenchymal cells in gliomas. The finding that t-PA was present in brain vessels adjacent to tumour indicates that its production is an intrinsic property of endothelium which is enhanced when it is stimulated to proliferate by tumour angiogenesis factors. A purified fibroblast growth factor-like substance derived from a human hepatoma cell line has recently been shown to promote angiogenesis *in vivo* and induce PA activity, enhance DNA synthesis and promote motility in confluent bovine endothelial cells (Presta *et al*., 1986). Lymphokines have also been shown to have a similar inductive effect on endothelium (Tiku & Tomasi, 1985). These observations provide support for the view that the phenotypically transformed t-PA and FN producing cells derived *in vitro* from human malignant gliomas are of vascular origin and are responding to factors produced by neoplastic glial cells (Manoury, 1977; Franks & Burrow, 1986; Jacobsen *et al*., 1987; McKeever *et al*., 1987; Rutka *et al*., 1987). Clearly a broader study of endothelium in other sites (neoplastic, reactive and normal) and the response of cultured cells to glioma-derived factors would be of interest.

The consistent failure of the vessels of low grade astrocytomas to show t-PA activity raises the possibility that this phenomenon could be utilised in grading of these tumours, although this would clearly need examination of larger numbers of cases and of intermediate forms.

Vascularity of malignant gliomas, and in particular aberrant vascular proliferation, has been found to correlate with a poorer prognosis (Cohadon *et al*., 1985; Fulling & Garcia, 1985) and there might be profit in attempting to correlate t-PA activity with outcome.

Although this study did not set out to examine the value of Uel reactivity in the diagnosis of tumours, the pattern observed in the three metastases is of interest in the light of the observation that staining of breast carcinomas with Uel correlates with outcome (Fenlon *et al*., 1987). The possible value of Uel staining in the diagnosis of central nervous system metastases and their distinction from anaplastic gliomas might repay further study using both cryostat sections and paraffin processed tissue.

We are grateful to Dr Keith Bradbury (Leeds) and Dr Jeanne Bell (Edinburgh) for helpful advice and criticism, to Steve Toms for photographic assistance, to Fraser Lewis for help with the Western blot and to Mr Keith James in the Department of Surgery, Edinburgh University for the gift of antibody. This project was supported by a grant from the special trustees, Leeds General Infirmary.

### References

ALI, S.Y. & LACK, C.H. (1965). Studies on the tissue activator of plasminogen. Distribution of activator and proteolytic activity in the subcellular fractions of rabbit kidney. *Biochem. J.*, **96**, 63.

COHADON, F., AOUAD, N., ROUGIER, A., VITAL, C., RIVEL, J. & DARTIGUES, J.F. (1985). Histologic and non-histologic factors correlated with survival time in supratentorial astrocytic tumours. *J. Neuro-oncol.*, **3**, 105.

FENLON, S., ELLIS, I.O., BELL, J., TODD, J.H., ELSTON, C.W. & BLANEY, R.W. (1987). Helix pomatia and *Ulex europaeus* lectin binding in human breast carcinoma. *J. Pathol.*, **152**, 169.

FRAME, M.C., FRESHNEY, R.I., VAUGHAN, P.F.T., GRAHAM, D.I. & SHAW, R. (1984). Inter-relationship between differentiation and malignancy-associated properties in glioma. *Br. J. Cancer*, **49**, 269.

FRANKS, A.J. & BURROW, H.M. (1986). *In vitro* heterogeneity in human gliomas. Are all transformed cells of glial origin? *Anticancer Res.*, **6**, 625.

FULLING, K.H. & GARCIA, D.M. (1985). Anaplastic astrocytoma of the adult cerebrum. Prognostic value of histologic features. *Cancer*, **55**, 928.

JACOBSEN, P.F., JENKYN, D.Y. & PAPADIMITRIOU, J.M. (1987). Four permanent cell lines established from human malignant gliomas: Three exhibiting striated muscle differentiation. *J. Neuropathol. Exp. Neurol.*, **46**, 431.

KENNEDY, P., WATKINS, B., THOMAS, D. & NOBLE, M. (1987). Antigenic expression by cells derived from human gliomas does not correlate with morphological classification. *Neuropathol. Appl. Neurobiol.*, **13**, 327.

KOHGA, S., HARVEY, S.R., WEAVER, R.M. & MARKUS, G. (1985). Localisation of plasminogen activators in human colon cancer by immunoperoxidase staining. *Cancer Res.*, **45**, 1787.

LAUG, W.E., TOKES, Z.A., BENEDICT, W.F. & SORGENTE, N. (1980). Anchorage independent growth and plasminogen activator production by bovine endothelial cells. *J. Cell Biol.*, **84**, 281.

LOLAIT, S.J., HARMER, J.H., AUTERI, G., PEDERSEN, J.S. & TOH, B.H. (1983). Expression of glial fibrillary acidic protein, actin, fibronectin, and factor VIII antigen in human astrocytomas. *Pathology*, **15**, 373.

McAUSLAN, B.R., HANNAN, G.N., REILLY, W. & STEWART, F.H.C. (1980). Variant endothelial cells. Fibronectin as a transducer of signals for migration and neovascularisation. *J. Cell. Physiol.*, **104**, 177.

McKEEVER, P.E., SMITH, B.H., TAREN, J.A., WAHL, R.L., KORNBLITH, P.L. & CHRONWALL, B.M. (1987). Products of cells cultured from gliomas. VI. Immunofluorescent, morphometric, and ultrastructural characterization of two different cell types growing from explants of human gliomas. *Am. J. Pathol.*, **127**, 358.

McLEAN, J.S., FRAME, M.C., FRESHNEY, R.I., VAUGHAN, P.F.T., MACKIE, A.E. & SINGER, I. (1986). Phenotypic modification of human glioma and non-small cell lung carcinoma by glucocorticoids and other agents. *Anticancer Res.*, **6**, 1101.

MANOURY, P. (1977). Establishment and characterisation of 5 human cell lines derived from a series of 50 primary intracranial tumours. *Acta Neuropathol.*, **39**, 33.

MARKUS, G., KOHGA, S., CAMIOLO, S.M., MADEJA, J.M., AMBRUS, J.L. & KARAOUSIS, C. (1984). Plasminogen activators in human malignant melanoma. *JNCI*, **72**, 1213.

MULLINS, D.E. & RORHLICH, S.T. (1983). The role of proteinases in cellular invasiveness. *Biochim. Biophys. Acta*, **695**, 177.

OGSTON, D. (1983). *The Physiology of Haemostasis*. Croom Helm: London.

PAETAU, A. (1988). Glial fibrillary acidic protein, vimentin and fibronectin in primary cultures of human glioma and fetal brain. *Acta Neuropathol.*, **75**, 448.

PRESTA, M., MOSCATELLI, D., JOSEPH-SILVERSTEIN, J. & RIFKIN, D.B. (1986). Purification from a human hepatoma cell line of a basic fibroblast growth factor-like molecule that stimulates capillary endothelial cell plasminogen activator production, DNA synthesis, and migration. *Molec. Cell. Biol.*, **6**, 4060.

QUIGLEY, J.P. (1976). Association of a protease plasminogen activator with a specific membrane fraction isolated from transformed cells. *J. Cell. Biol.*, **71**, 472.

QUINDLEN, E.A. & BUCHER, A.P. (1987). Correlation of tumour plasminogen activator with peritumoral edema. *J. Neurosurg.*, **66**, 729.

REDDY, K.N.N. & KLINE, D.L. (1980). Plasminogen activators. In *Fibrinolysis*, Kline, D.L. & Reddy, K.N.N. (eds) ch. 2. CRC Press: Boca Raton, Florida.

RIJKEN, D.C. & COLLEN, D. (1981). Purification and characterisation of the plasminogen activator secreted by human melanoma cells in culture. *J. Biol. Chem.*, **256**, 7035.

RIJKEN, D.C., WIJNGAARDS, G. & WELBERGEN, J. (1980). Relationship between tissue plasminogen activator and the activators in blood and vascular wall. *Thromb. Res.*, **18**, 815.

RIJKEN, D.C., WIJNGAARDS, G., ZAAL-DE JONG, M. & WELBERGEN, J. (1979). Purification and partial characterisation of plasminogen activator from human uterine tissue. *Biochim. Biophys. Acta*, **580**, 140.

RUTKA, J.T., GIBLIN, J.R., DOUGHERTY, D.Y. & 6 others (1987). Establishment and characterization of five cell lines derived from human malignant gliomas. *Acta Neuropathol.*, **75**, 92.

TAKASHIMA, S., KOGHA, M. & TANAKA, K. (1969). Fibrinolytic activity of human brain and cerebrospinal fluid. *Br. J. Exp. Pathol.*, **50**, 533.

TIKU, M.L. & TOMASI, T.B. (1985). Enhancement of endothelial plasminogen activator synthesis by lymphokines. *Transplantation*, **40**, 293.

TODD, A.S. (1959). The histological localisation of fibrinolysin activator. *J. Pathol. Bacteriol.*, **78**, 281.

TOWBIN, H., STAEHELIN, T. & GORDON, J. (1979). Electrophoretic transfer of proteins from polyacrylamide gels to nitrocellulose sheets: Procedure and some applications. *Proc. Natl Acad. Sci. USA*, **76**, 4350.

TRIPATHI, B.J., GEANON, J.D. & TRIPATHI, R.C. (1987). Distribution of tissue plasminogen activator in human and monkey eyes. *Ophthalmology*, **94**, 1434.

TUCKER, W.S., KIRSH, W.M., MARTINEZ-HERNANDEZ, A. & FINK, L.M. (1978). *In-vitro* plasminogen activator activity in human brain tumours. *Cancer Res.*, **38**, 297.

UNKELESS, J.C., GORDON, S. & REITCH, E. (1974). Secretion of plasminogen activator by stimulated macrophages. *J. Exp. Med.*, **139**, 834.